# Germline-Restricted Chromosome (GRC) in Female and Male Meiosis of the Great Tit (Parus major, Linnaeus, 1758)

**DOI:** 10.3389/fgene.2021.768056

**Published:** 2021-10-25

**Authors:** Anna Torgasheva, Lyubov Malinovskaya, Kira Zadesenets, Elena Shnaider, Nikolai Rubtsov, Pavel Borodin

**Affiliations:** ^1^ Institute of Cytology and Genetics, Russian Academy of Sciences, Siberian Branch, Novosibirsk, Russia; ^2^ Novosibirsk State University, Novosibirsk, Russia; ^3^ Bird of Prey Rehabilitation Centre, Novosibirsk, Russia

**Keywords:** avian chromosomes, programmed DNA elimination, recombination, synaptonemal complex, MLH1, SYCP3, crossing over

## Abstract

All songbirds studied so far have a germline-restricted chromosome (GRC), which is present in the germ cells and absent in the somatic cells. It shows a wide variation in size, morphology, and genetic content between the songbird species. In this paper, we analyzed GRC behavior in female and male meiosis of the great tit, using immunolocalization of meiotic proteins and FISH with GRC-derived DNA probes. We found that, despite dozens of million years of independent evolution, the great tit GRC displays a striking similarity with the GRCs of two species of martins and two species of estrildid finches examined earlier. It was usually present in two copies in females forming recombining bivalent and in one copy in males forming a condensed heterochromatic body with dotted-like axial elements of the synaptonemal complex. We observed mosaicism for the GRC copy number in the female and male great tit. This indicates that one of the GRC copies might be passively lost during premeiotic germ cell divisions. After the meiotic prophase, the GRC was ejected from most male germ cells. The reverse and interspecies FISH with GRC-specific microdissected DNA probes indicates that GRCs of the great tit, pale martin, and zebra finch differ substantially in their genetic content despite similarities in the meiotic behavior.

## Introduction

Programmed DNA elimination has been observed in many species of different taxa (Wang and Davis, 2014). One of the most recent examples is the germline-restricted chromosome (GRC) of the songbirds, which is present in the germline and absent in the somatic cells (Pigozzi and Solari, 1998, Pigozzi and Solari, 2005; del Priore et al., 2014; Kinsella et al., 2019; Torgasheva et al., 2019; Malinovskaya et al., 2020). In the male germ cells, it is usually present in one copy. It is heterochromatic, highly enriched in histone H3 trimethylated at lysine 9, and ejected from the nuclei after the meiotic divisions (del Priore et al., 2014). In the female germline, the GRC is usually present in two copies, which pair and recombine with each other and are transmitted to the progeny (Pigozzi and Solari, 2005; Torgasheva et al., 2019; Malinovskaya et al., 2020).

There is a variation in GRC copy number in three species examined: zebra finch, sand martin, and pale martin. Most oocytes of zebra finches and martins contained two copies of GRC, but some specimens had one copy (Pigozzi and Solari, 2005; Torgasheva et al., 2019; Malinovskaya et al., 2020). Male pale martins demonstrated mosaicism for the number of GRCs in the spermatocytes. Most cells contained one copy, but the cells with two and three copies were also detected (Malinovskaya et al., 2020). More species have to be examined to estimate an abundance and possible causes of the GRC polymorphism and mosaicism.

The GRC shows a wide variation in size, morphology, and genetic content between the songbird species. In most species, it is a large macrochromosome. In other species, it is a microchromosome (Torgasheva et al., 2019). Cross-species fluorescent *in situ* hybridization (FISH) with GRC-derived DNA probes revealed low, if any, homology between GRCs of distantly related species (Torgasheva et al., 2019). The intraspecies variation in the number of GRCs in the germ cells and its interspecies variation in size and genetic content are intriguing because GRC appears to be an indispensable component of the germ cells. Detailed analysis of zebra finch GRC revealed that it contains dozens of genes actively transcribed in the germ cells. Some of them show signs of positive selection (Biederman et al., 2018; Kinsella et al., 2019).

In this paper, using immunolocalization of several meiotic proteins, we examined GRC behavior in female and male meiosis of the great tit (*Parus major* Linnaeus, 1758) and compared it with that of two estrildid finches (zebra and Bengalese) (Pigozzi and Solari, 1998, Pigozzi and Solari, 2005; del Priore et al., 2014) and two martins (sand and pale) (Malinovskaya et al., 2020). We also estimated a homology between the GRC of these species using cross-species FISH.

## Materials and Methods

### Experimental Model

We examined seven adult males and seven female nestlings collected in two mixed forest parks in Novosibirsk (54,50N; 83,05E and 55.09N, 82.95E). The males were captured with bird nets at the beginning of the breeding season. Nestling females on days 3–6 after hatching were collected from the nests.

### Conventional Chromosome Spreading and Staining

Mitotic chromosome spreads were prepared from short-term bone marrow cell cultures incubated in Dulbecco’s modified Eagle’s medium (ThermoFisher Scientific, cat# 41965062) with 10 μg/ml colchicine (Sigma, cat# C9754) for 2 h at 37°C. The cells were swollen in 0.56% KCl, fixed in fresh Carnoy’s solution (methanol:glacial acetic acid, 3:1). The cell suspension was dropped on clean, cold, wet microscope slides and spread by air-drying. Chromosomes were stained with DAPI dissolved in Vectashield antifade solution (Vector Laboratories, cat# H-1200-10, United States).

Meiotic chromosome spreads were prepared from a suspension of testicular cells of adult males treated with hypotonic solution (0.88% KCl) for 3 h at 37°C and then with Carnoy’s solution. The cell suspension was dropped onto clean, cold, wet coverslips (60 × 24 mm, 0.17 mm thick), and dried. Chromosomes were stained with 0.1% Giemsa solution (Biovitrum, cat# 20-043\L).

### Synaptonemal Complex Spreading and Immunostaining

Chromosome spreads for SC analysis were prepared from testes and ovaries by the drying down method (Peters et al., 1997). Testes and ovaries were dissected and placed for 30–60 min in an extraction buffer containing 30 mM Tris (Helicon, cat# 77-86-1, Russia), 50 mM sucrose (Sigma, cat# S7903-1KG), 17 mM trisodium citrate dehydrate (Chimmed, cat# A1227436-500.0, Russia), 5 mM EDTA (Panreac&AppliChem, cat# A5097), and pH 8.2. Then, small pieces of testis or the whole ovary were macerated in 40 µl of 100 mM of sucrose, pH 8.2, on a glass slide. A fine suspension was made, and 20 µl of the suspension was gently dropped at the slide moistened by 1% paraformaldehyde (Sigma-Aldrich, cat# 158127) solution, pH 9.2, containing 0.15% Triton X-100 (Sigma, cat# T8787). The slides were dried for 2 h, washed in 0.4% Kodak Photo-Flo 200 (Kodak, cat# 742057), and dried at room temperature.

Immunostaining was performed according to Anderson et al. (1999) using rabbit polyclonal anti-SYCP3 (1:500; Abcam, cat# ab15093), mouse monoclonal anti-MLH1 (1:50; Abcam, cat# ab14206), human anticentromere (ACA) (1:100; Antibodies Inc., cat# 15–234), and rabbit polyclonal anti-H3K9me3 (1:50; Abcam, cat# ab8898) primary antibodies. The secondary antibodies used were Cy3-conjugated goat antirabbit (1:500; Jackson ImmunoResearch, cat# 111-165-144), FITC-conjugated goat antimouse (1:50; Jackson ImmunoResearch, cat# 115-095-003), and AMCA-conjugated donkey antihuman (1:100; Jackson ImmunoResearch, cat# 709-155-149). Antibodies were diluted in PBT [3% bovine serum albumin and 0.05% Tween 20 in phosphate-buffered saline (PBS)]. Slides were incubated in a solution of 10% PBT for 40 min to reduce the unspecific binding of the antibodies. Primary antibody incubations were performed overnight in a humid chamber at 37°C and secondary antibody incubations for 1 h at 37°C. After antibody incubations, slides were washed three times in PBST (PBS with 0.1% Tween 20) for 10 min. Slides were mounted in Vectashield antifade mounting medium (Vector Laboratories, cat# H-1000-10, United States).

### Generation of the Microdissected DNA Probe and FISH

The DNA probe for the great tit GRC was prepared by microdissection of 15 round Giemsa-positive bodies located near spermatocytes and spermatids at the conventionally prepared meiotic chromosome spreads. According to Pigozzi and Solari (1998) they contain GRC ejected from the germ cells. DNA isolated from the microdissected material was amplified with the GenomePlex Single Cell Whole Genome Amplification Kit (Sigma-Aldrich, cat# WGA4) according to the manufacturer’s protocol (Zadesenets et al., 2017). The obtained PCR products were labeled with Flu-dUTP (Genetyx, cat# N-801000, Russia) in additional PCR cycles. DNA probes for zebra finch and pale martin GRCs were prepared as described earlier (Torgasheva et al., 2019). C_0_t-1 DNA isolation from pale martin was performed as described earlier (Trifonov et al., 2017).

FISH with the DNA probes on the SC spreads were performed according to a standard protocol with salmon sperm DNA (Ambion, cat# AM9680, United States) as a DNA carrier (Trifonov et al., 2017). Chromosomes were counterstained with DAPI dissolved in Vectashield antifade solution.

### Microscopic Analysis

Images of DAPI-stained metaphase chromosomes and SC spreads after immunostaining and FISH were captured using a CCD camera installed on an Axioplan 2 compound microscope (Carl Zeiss, Germany) equipped with filter cubes #49, #10, and #15 (ZEISS, Germany) using ISIS4 (METASystems GmbH, Germany).

### Chromosome Measurements and Generation of Recombination Maps of GRCs

Centromeres were identified by ACA foci. MLH1 signals were only scored if they were localized on SCs. The length of the SC was measured in micrometers, and the positions of MLH1 foci in relation to the centromere were recorded using MicroMeasure 3.3 (Reeves, 2001). SCs of GRC and macrochromosomes were identified by their relative lengths and centromeric indices. STATISTICA 6.0 software package (StatSoft, Tulsa, OK, United States) was used for descriptive statistics. All results were expressed as mean ± SD.

## Results

### Pachytene Karyotype of the Great Tit

**FIGURE 1:**
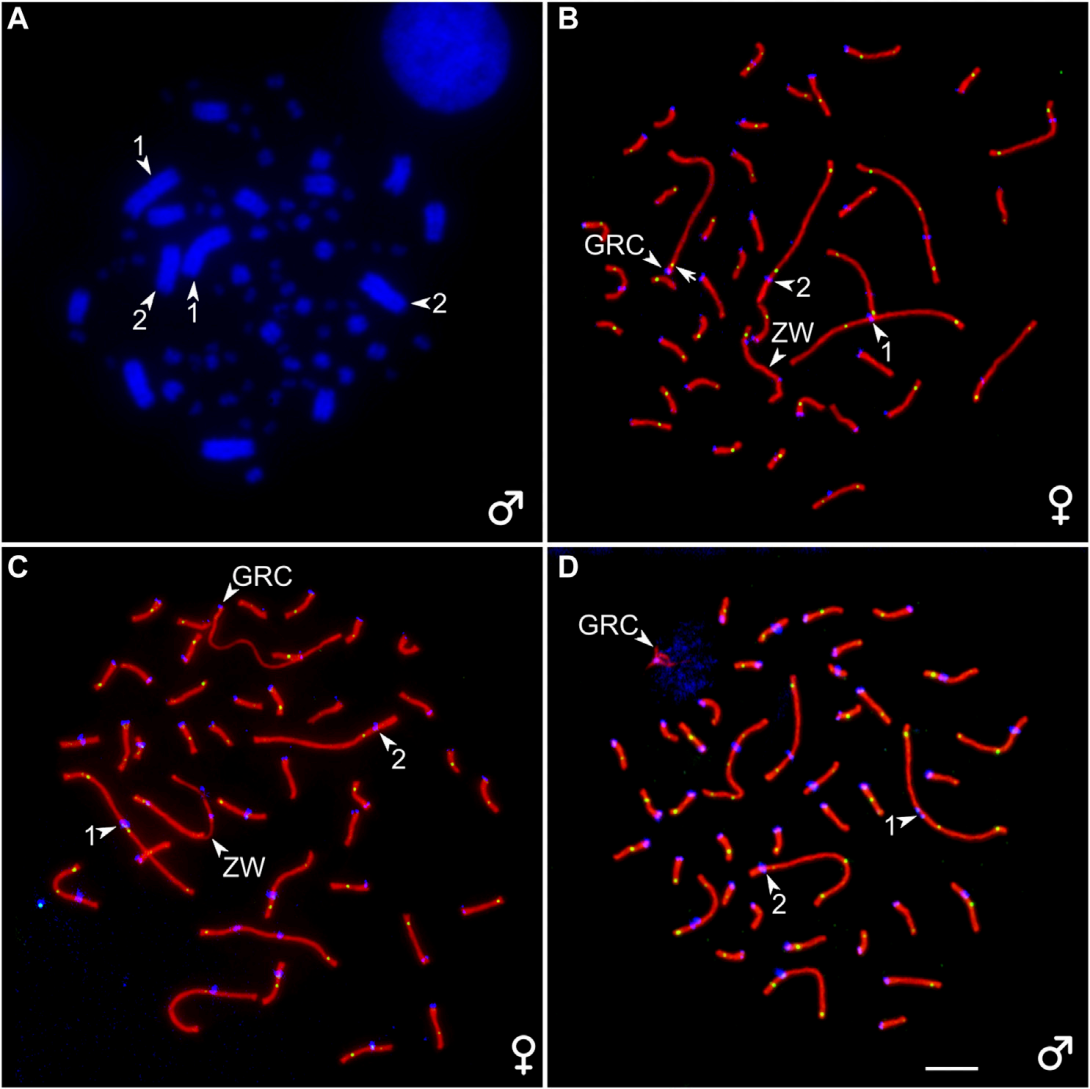
**(A)** DAPI-stained bone marrow cell of the male great tit. **(B–D)** Pachytene oocytes **(B, C)** and spermatocyte **(D)** of the great tit with two **(B)** and one **(C, D)** copies of GRC after immunostaining with antibodies to SYCP3 (red), centromere proteins (blue), and MLH1 (green). The arrowheads point to centromeres of the two largest macrobivalents and ZW bivalent (identified by heteromorphic SC and unaligned centromeres) and GRC. The arrow points to the MLH1 signal at GRC bivalent **(B)**. Bar–5 μm.

A diploid chromosome number (2n) in the somatic cells of the great tit was 80 and corresponded to that previously described (Nanda et al., 2011). Macrochromosome 1 was metacentric; 2 was subacrocentric; 3, 4, 5, and 6 were submetacentric; and all other chromosomes but five were telocentric, forming a row gradually decreasing in length. Z chromosome was a submetacentric macrochromosome of intermediate size. The W chromosome was a large microchromosome. Pachytene karyotype contained six macrobivalents, 33 microbivalents, the sex bivalent (ZZ in males, ZW in females), and an additional bivalent or univalent of a large acrocentric chromosome, which was not present on the bone marrow spreads (Figure 1). We identify this chromosome as a GRC.

### Mosaicism for GRC Copy Number in Females

We detected mosaicism for the number of GRC copies in pachytene oocytes (Supplementary Table 1). Three out of seven females contained two copies of GRC in all examined cells at the pachytene stage (Figure 1B). Four females were mosaic for GRC copy number. Most of their oocytes contained two GRCs. The proportion of cells with one GRC varied from 2 to 26% (Supplementary Table 1). The GRC bivalents appeared as normal autosomal bivalents with at least one MLH1 focus (Figure 1B) and were distinguished as the only acrocentrics. The GRC univalents were distinguished from the bivalents by a lack of MLH1 signals and less intense labeling with antibodies to SYCP3 (Figure 1C). They were significantly longer than the bivalents (19.4 ± 3.0 and 14.1 ± 5.4 µm, respectively; Mann–Whitney test, *p* = 0.02).

### Synapsis and Recombination of GRC in Females

GRC bivalents differed substantially in average number and distribution of MLH1 foci from the macrobivalents of comparable size (SC1: 17.2 ± 3.7 µm and SC2: 14.7 ± 3.1 µm). Most of the GRC bivalents contained one MLH1 signal. It was always located near the centromere (Supplementary Image 1). The bivalents with two or three MLH1 signals were rare (4.0 and 0.4%, respectively). The average number of MLH1 signals per GRC bivalent was 1.05 ± 0.2. The macrobivalents 1 and 2 contained a significantly higher number of MLH1 foci (3.5 ± 0.9 and 2.7 ± 0.8, respectively, Mann–Whitney test, *p* = 0.00), which showed a rather even distribution with peaks near the bivalent ends (Supplementary Figure S1).

### Mosaicism for GRC Copy Number in Males

In total, we analyzed 612 spermatocytes from seven males of the great tit. In all analyzed cells, GRC occurred as a condensed body extensively labeled by antibodies to the centromere proteins. SYCP3 signal was only observed near the proximal end of GRC or its both ends as single or double dots or short lines (Figure 1D).

To estimate the copy number variation of GRC at different stages of spermatogenesis, we used antibodies against histone H3 trimethylated at lysine 9 (H3K9me3). It is a marker of heterochromatic transcriptionally repressed regions (Nicetto and Zaret, 2019). The zebra finch GRC is enriched in H3K9me3 compared with the basic chromosome set during prophase I and after the elimination (del Priore et al., 2014).

**FIGURE 2:**
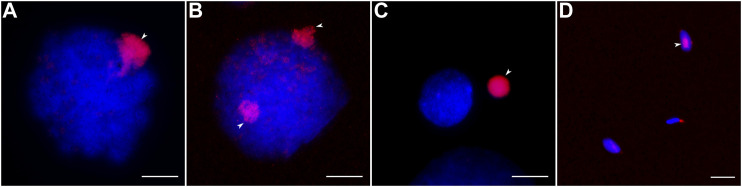
Male germ cells after H3K9me3 labeling (red) and DAPI staining (blue). **(A, B)** Cells with one **(A)** and two **(B)** GRC copies. **(C)** Post-meiotic cell and eliminated GRC. **(D)** Spermatozoa with and without GRC. Arrowheads point to GRCs. Bar—10 µm.

Most male germline cells of the great tit contain one strong H3K9me3 signal marking the GRC (Figure 2A). In 15 cells out of the 411 examined (3.6%) we detected two H3K9me3 signals (Figure 2B). Spermatids and spermatozoa usually do not show the H3K9me3 signals. Near some of these cells, we detected condensed chromatin bodies with strong H3K9me3 signal (Figure 2C). Apparently, they were GRCs ejected from the cells. Surprisingly, we found the H3K9me3 signal in a few spermatozoa (3 out of 880 cells examined) (Figure 2D).

### FISH With the GRC-Specific DNA Probe

**FIGURE 3:**
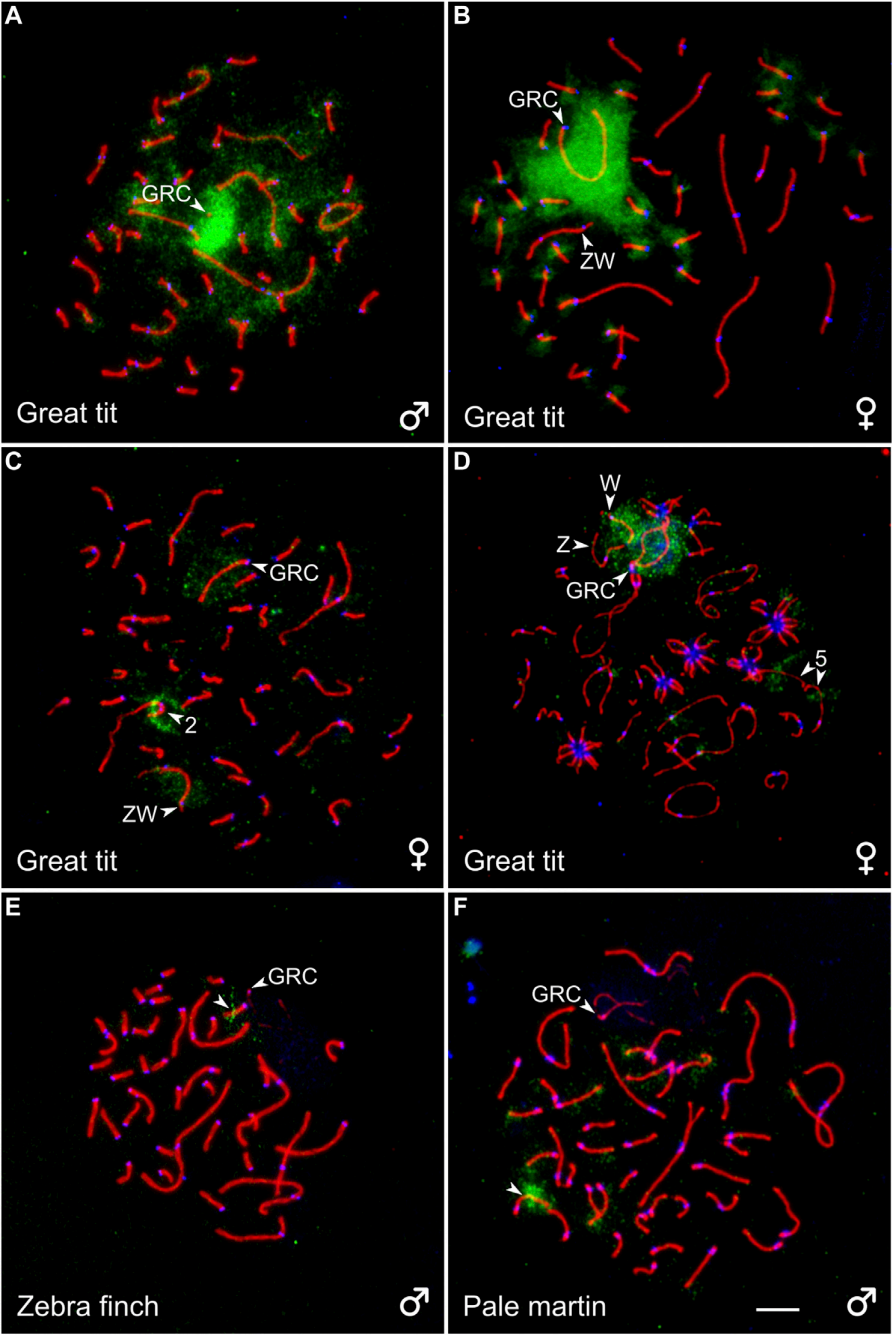
SC spreads of the great tit **(A–D)**, zebra finch **(E)**, and pale martin **(F)** after reverse FISH with the great tit GRC probe (green) without **(A)** and with **(B)** suppression of repetitive sequences using C_0_t-1 DNA of the great tit and cross-species FISH with DNA probes (green) derived from GRC of the zebra finch **(C)**, pale martin **(D)**, and great tit **(E, F)**. Spreads were immunolabeled with antibodies against SYCP3 (red) and centromere proteins (blue). The arrowheads point to macrobivalents 2 and 5, sex bivalent ZW, GRC and microbivalents with hybridization signals. Bar—5 µm.

To estimate a homology between GRC and the chromosomes of the basic set of the great tit, we performed reverse FISH with the GRC DNA probe. It produced a strong specific signal on the condensed GRC in pachytene spermatocytes (Figure 3A). It also faintly labeled different regions of chromosomes of the basic set. In pachytene oocytes, FISH with suppression of repeated sequences using C_0_t-1 DNA produced a strong specific signal on the whole GRC except short pericentromeric region (Figure 3B), where most MLH1 foci were located (Supplementary Image 1). It also labeled pericentromeric regions of most microchromosomes (Figure 3B). We did not detect GRC probe signals in post-meiotic cells.

To estimate a homology between GRC of different species, we carried out cross-species FISH of the zebra finch and pale martin GRC DNA probes with the great tit oocytes and the great tit GRC DNA probe with the zebra finch and pale martin spermatocytes (Figures 3C–F). The zebra finch and pale martin GRC probes produced a clear hybridization signal at the GRC of the great tit. They also faintly labeled its W chromosome (Figures 3C,D). Additionally, the zebra finch GRC DNA probe produced a distinct signal at the short arm of the SC2 (Figure 3C) and the pale martin GRC DNA probe in the middle of the long arm of the SC5 (Figure 3D). The great tit GRC probe gave a strong signal at the middle of one zebra finch microbivalent and the distal half of one pale martin microbivalent (Figures 3E,F). We also found the signals of the great tit GRC probe at telomeres of some macro- and microbivalents of pale martin. We did not detect signals of great tit GRC probe at zebra finch and pale martin GRCs.

## Discussion

The estimated times of divergence of the great tit from estrildid finches and martins are approximately the same: 38 MYA CI: (34–42 MYA) and 40 MYA CI: (37–43 MYA), respectively (Claramunt and Cracraft, 2015). Our results of cross-species FISH with GRC DNA probes of these species indicate that GRCs of each pair of these three phylogenetically equidistant species of songbirds still share some common (probably repetitive) sequences. In reverse FISH, the great tit GRC probe labeled pericentromeric regions of microchromosomes. Torgasheva et al. (2019) observed a similar effect in the reverse FISH experiment in the pale martin. This indicates GRCs of these species are enriched with repeated sequences characteristic to pericentromeric regions. However low intensity of the cross-species hybridization signals and lack of hybridization signal of great tit GRC probe at zebra finch and pale martin GRCs suggest that GRCs of the zebra finch, pale martin, and great tit have already undergone substantial genetic divergence. The distribution of GRC probe FISH signals on chromosomes of the basic set also confirms significant differences in genetic content between GRCs of these species.

Yet, despite dozens of million years of independent evolution and a substantial divergence in the genetic content, GRCs of the great tit, estrildid finches, and martins are very similar in their morphology and meiotic behavior. They are large acrocentric macrochromosomes of approximately the same size (Pigozzi and Solari, 1998; del Priore et al., 2014; Malinovskaya et al., 2020). The great tit, estrildid finches, and martins show the same sexual dimorphism in the GRC copy number. Most males examined had one GRC in spermatocytes; all females had two GRCs in the majority of their oocytes (Pigozzi and Solari, 2005; Malinovskaya et al., 2020).

Recombination in the GRC bivalents of the great tit, zebra finch, and pale and sand martins is strongly suppressed everywhere beyond the chromosome ends. The only difference is that the GRC bivalents of the great tit usually contain a single recombination nodule located in their pericentromeric region, and the GRCs of the finches and martins have two nodules at their both ends (Pigozzi and Solari, 2005; Malinovskaya et al., 2020). Malinovskaya et al. (2020) suggest that the polarized recombination pattern along the GRC bivalents in the female songbirds could facilitate GRC non-disjunction during MI. It is shown that in human oocytes chiasmata located too close to centromere are responsible for a high rate of non-disjunction in female meiosis (Hassold and Hunt, 2001).

In all species examined, we observed sexual dimorphism in the appearance of GRC univalents. In female meiosis, it appears as a normal lateral element of SC, and in males its lateral element is usually thin and often fragmented (Pigozzi and Solari, 1998; del Priore et al., 2014; Torgasheva et al., 2019; Malinovskaya et al., 2020). The GRC univalents in great tit males show weak or no polymerization of the lateral elements at all. This might indicate a greater degree of GRC heterochromatinization in this species.

The frequency of polymorphism and mosaicism for GRC copy number in songbirds is difficult to estimate because it would demand large samples of birds and germ cells. The data obtained to date indicate that the mosaicism is rather frequent in the martin males, rare in both sexes of the great tit, and has not been detected yet in the estrildid finches. However, the mere existence of the polymorphism and mosaicism for GRC elucidates two important features of GRC.

Polymorphism indicates that at least one GRC copy is indispensable for the germ cell survival until the MI in males and to term in females because no cells without GRC were observed in any species examined (Torgasheva et al., 2019). At the same time, the cells with one and two copies apparently have the same chances to survive. Mosaicism in males indicates that the GRC is actively ejected from spermatocytes after the meiotic prophase, but any additional copy of GRC can be passively lost during the germ cell mitotic divisions. Mosaicism in females indicates a possibility of the same passive loss of the second GRC during premeiotic germ cell divisions. This is consistent with the model of GRC transmission proposed earlier (Malinovskaya et al., 2020).

## Data Availability

The original contributions presented in the study are included in the article/Supplementary Material, further inquiries can be directed to the corresponding author.
