# Supplementary material for: Germline-Restricted Chromosome (GRC) in Female and Male Meiosis of the Great Tit (Parus major, Linnaeus, 1758)
Source: Front Genet. 2021 Oct 25;12:768056. doi: 10.3389/fgene.2021.768056 (PMC8573160; doi:10.3389/fgene.2021.768056)
Supplement: Supplementary file 1 [file Table1.pdf]

Supplementary Table 1.

Mosaicism for GRC copy number in oocytes of great tits

Specimen ID	Number of cells examined	Number of cells with one GRC	Number of cells with two GRC
1	65	0	65
2	88	0	88
3	89	0	89
4	50	1	49
5	54	6	48
6	27	7	20
7	93	9	84
Total	424	13	411
