# Supplementary material for: Germline-Restricted Chromosome (GRC) in Female and Male Meiosis of the Great Tit (Parus major, Linnaeus, 1758)
Source: Front Genet. 2021 Oct 25;12:768056. doi: 10.3389/fgene.2021.768056 (PMC8573160; doi:10.3389/fgene.2021.768056)
Supplement: Supplementary file 3 [file Image1.pdf]

Supplementary Figure 1

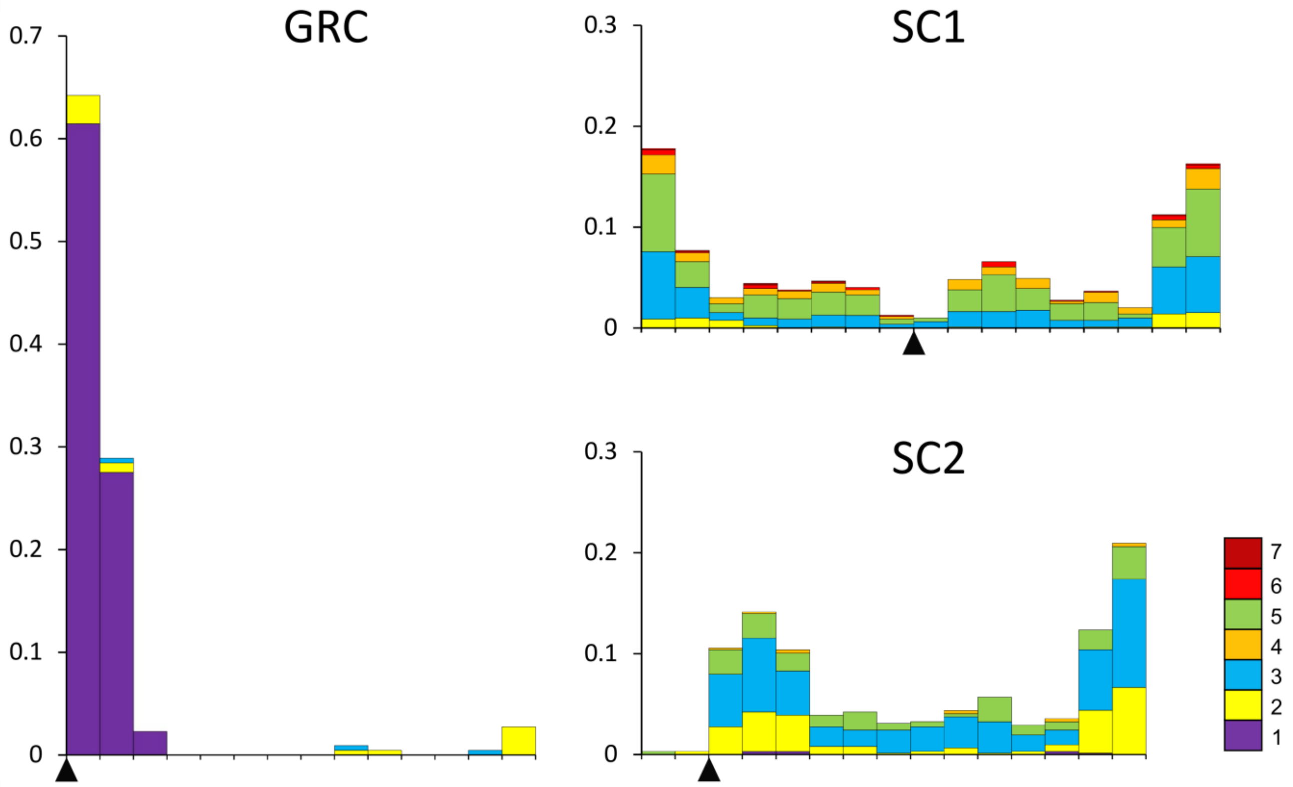

Supplementary Figure 1. Distribution of MLH1 signals along the GRC bivalent and two largest macrobivalents (SC1 and SC2) in pachytene oocytes of the great tit. The X-axis shows the position of MLH1 foci at the bivalent relative to the centromere (indicated by a triangle). The width of the interval is approximately one μm. The Y-axis reflects the proportion of bivalents with MLH1 foci in each interval. The colors represent the proportion of bivalents with one to seven MLH1 foci per bivalent.
